# Fetal thoracoamniotic shunting for severe macrocystic congenital pulmonary airway malformation with the Somatex^®^ intrauterine shunt: intrauterine course and postnatal outcome

**DOI:** 10.1007/s00404-025-08247-5

**Published:** 2026-01-20

**Authors:** C. Oelgeschläger, E. C. Weber, I. Gottschalk, J. Jimenez-Cruz, A. Geipel, B. Strizek, J. Kohaut, M. Dübbers, C. Oetzmann von Sochaczewski, A. Heydweiller, C. Berg

**Affiliations:** 1 Division of Prenatal Medicine, Gynecological Ultrasound and Fetal Surgery, Department of Obstetrics, University Hospital Cologne, University of Cologne, Kerpener Strasse 34, 50931 Cologne, Germany; 2 Department of Obstetrics and Prenatal Medicine, University Hospital Bonn, Bonn, Germany; 3 Division of Pediatric Surgery, Faculty of Medicine and University Hospital Cologne, University of Cologne, Cologne, Germany; 4 Sektion Kinderchirugie der Chirugischen Klinik, Universitätsklinikum Bonn, Bonn, Germany

**Keywords:** Congenital malformation of the lung, CPAM, Fetal thoracoamniotic shunting, Somatex^®^ intrauterine shunt

## Abstract

**Purpose:**

Thoracoamniotic shunting (TAS) in fetuses with macrocystic congenital pulmonary airway malformation (CPAM) is mostly performed with pigtail shunts like the rocket shunt or the Harrison fetal bladder stent. The aim of this study was to assess the prenatal course, perinatal outcome and complications of TAS for severe macrocystic CPAM using the Somatex^®^ intrauterine shunt.

**Methods:**

This was a two center (Cologne/Bonn) observational retrospective study of fetuses that underwent TAS using the Somatex^®^ intrauterine shunt for severe macrocystic CPAM with and without hydrops between 2016–2024. Outcome parameters were perinatal survival, complications, gestational age at delivery and visibility of the shunt outside the skin after birth.

**Results:**

During the study period, 25 fetuses were treated with the Somatex^®^ shunt (13 = Cologne, 12 = Bonn), including 24 singletons and one fetus of a monochorionic-diamniotic twin pregnancy Mean gestational age at intervention was 24.7 weeks (range 19–30). The mean diameter of the dominant cyst within the lesion was 34 mm (range 18–55). Fetal hydrops prior to TAS (ascites and fetal scalp oedema) was present in 36% (9/25). Dislocation in the further course of pregnancy occurred in 8% (2/25) with the need for reintervention in two cases. Resolution of hydrops and regression of the lesion occurred in 96% (24/25). Mean gestational age at delivery was 38.3 weeks (range 26–41), the preterm birth rate < 37 weeks was 20% (5/25), 12% (3/25) were due to PPROM. Live birth rate was 100% and 92% (23/25) of neonates survived the neonatal period. Of the 12 liveborns delivered at the two study centers, in one case the shunt (8.3%) was dislocated in the amniotic cavity, 5 (41.7%) had a visible shunt outside the skin, whereas in the other 6 (50.0%) cases the shunt was covered with skin at birth.

**Conclusions:**

TAS in macrocystic CPAM with the Somatex^®^ shunt has a high technical success rate leading to high neonatal survival rates even in cases associated with hydrops. The intrauterine course and neonatal outcome are comparable to TAS for fetal macrocystic CPAM using other types of shunts. Therefore, the choice of the shunt in macrocystic CPAM can be made freely at the discretion of the physician in charge, the availability of devices and economic factors. Due to the short length of 25 mm and its straight design, the outer end of the Somatex^®^ shunt is covered by skin at birth in up to 50% of cases, which may complicate its removal.

## Take-home message

**Table:** 

The intrauterine course and the survival rates of fetal thoracoamniotic shunting for macrocystic CPAM with the Somatex^®^ intrauterine shunt are comparable to other types of shunts. Advantage of the Somatex^®^ shunt is the smaller size of the delivering cannula however due its short length und straight design it could be covered by skin and warrants surgical removement after birth.	

## Introduction

Large macrocystic congenital pulmonary airway malformations (CPAM) have a high risk of either causing pulmonary hypoplasia due to compression of lung tissue or development of hydrops due to compression of the heart [1]. Unlike microcystic lesions they rarely regress in the further course of pregnancy [2], mainly because of fluid accumulation in the cysts. It is assumed that even most microcystic lesions do not truly resolve, instead, they escape detection because the normal lungs become more echogenic in the third trimester [3]. Nevertheless, in the presence of hydrops, the prognosis is poor due to the risk of intrauterine demise and neonatal death. Up to 95% of hydropic fetuses with CPAM die before or after birth [4].

In 1987, the insertion of a thoracoamniotic shunt (TAS) for macrocystic CPAM was pioneered by Nicolaides et al. in a 24 week gestational age fetus [5, 6]. Nowadays, TAS is an established treatment for fetuses with macrocystic CPAM associated with hydrops and also in the absence of hydrops for very large lesions or lesions with a dominant cyst [7]. Studies of TAS for macrocystic CPAM with or without hydrops demonstrated an improved perinatal outcome [8–12].

The two most commonly used shunt systems for TAS are the Harrison Cook shunt and the Rocket shunt. But these shunt systems were developed nearly 40 years ago. Frequently reported problems of these shunts are dislocations and occlusion [8, 11].

Since late 2014, the Somatex^®^ shunt has been available in the European community. Whereas the Harrison and the Rocket shunt are delivered through a 13G cannula and fixed with pigtail ends, the Somatex^®^ shunt is delivered by a smaller 18G cannula and has self-deploying parasols at both ends to avoid dislocation. The Somatex^®^ shunt was initially designed for vesicoamniotic shunting (VAS). The dislocation rates of the Somatex^®^ shunt for VAS have been shown to be much lower (36% versus 87%) with improved perinatal survival compared to the Harrison shunt [13]. The Somatex^®^ shunt also showed a good performance in TAS for hydrothorax with less shunt-related complication rates, but with a higher rate of PROM [14].

We therefore evaluated the use of the Somatex^®^ intrauterine shunt in TAS for macrocystic CPAM and conducted a retrospective analysis of the intrauterine course and the perinatal outcome in two tertiary centers.

## Methods

This is a retrospective analysis of all cases with macrocystic CPAM that underwent shunting with the Somatex^®^ shunt (Somatex medical technologies Berlin, Germany) in two tertiary centers (university hospital of Cologne and university hospital of Bonn) during the period of 2016–2024.

Antenatal ultrasound reports and stored images were reviewed for CPAM size, size of the biggest cyst, laterality, presence of hydrops, mediastinal-shift and polyhydramnios. Other anomalies, such as bronchopulmonary sequestration or bronchogenic cysts, were excluded. There were no concomitant anomalies.

Diagnostic work-up prior to TAS included a complete ultrasound assessment of the fetus, including transabdominal fetal echocardiography and doppler sonography. As macrocystic CPAM is not regarded to be associated with structural chromosomal aberrations, conventional karyotyping was not considered essential. Fetal magnetic resonance imaging (MRI) was not performed as the diagnosis was certain in all cases.

Criteria for shunt intervention were hydrops or signs of evolving hydrops, such as ascites. Hydrops was defined as the accumulation of fluid in at least two fetal compartments. But also, non-hydropic fetuses with large lesions with severe mediastinal shift, large lesions with a dominant cyst, associated polyhydramnios, or growing lesions were considered candidates for shunting.

**Fig. 1:**
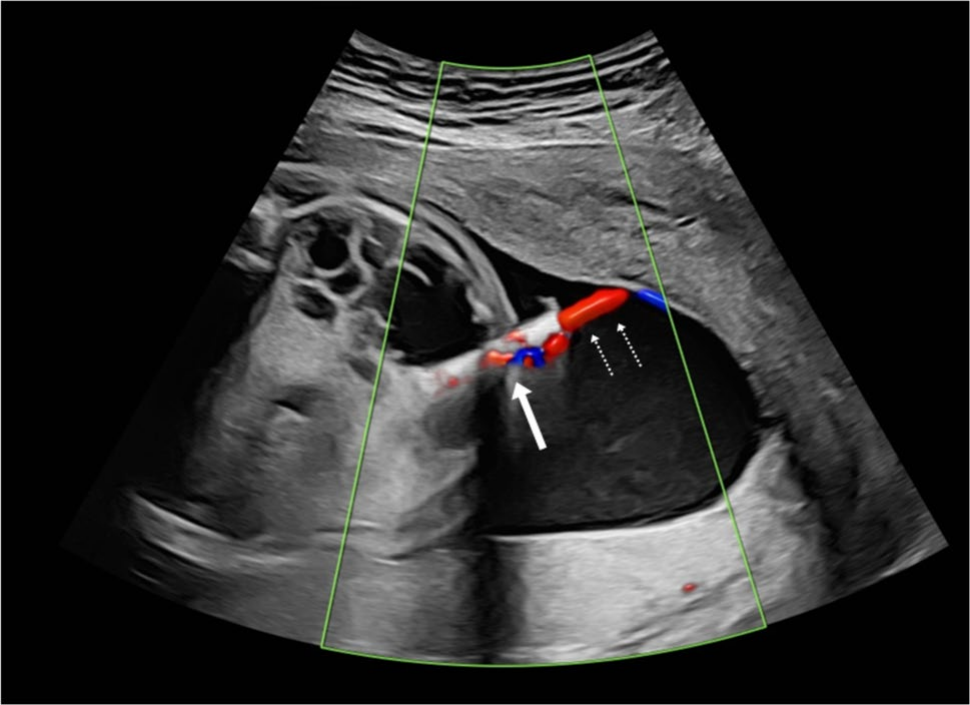
Somatex^®^ intrauterine shunt (arrow) seconds after placement in the largest cyst of a right sided CPAM at 26 weeks. The evacuation of fluid (dotted arrow) is demonstrated by color Doppler

The Somatex^®^ intrauterine shunt was inserted in all patients under ultrasound guidance as described in detail by Grandt et al. [14]. The most accessible of the larger cysts was shunted. The shunt was positioned with the inner end in the cyst and the outer end in the amniotic cavity (Fig. 1). For fetal anesthesia prior to shunting vecuronium (200 $\upmu$g/kg body weight) and fentanyl (10 $\upmu$g/kg body weight) was used. Fetal anesthesia was administered either by intravenous umbilical cord or via direct fetal intramuscular injection. No antibiotics or tocolytics were used. Some fetuses, especially in higher weeks of pregnancy with possible viability, received antenatal steroids for lung maturation. The shunt insertion was performed without maternal anesthesia.

All patients gave written informed consent for TAS after counseling. Follow-up scans were performed for the next 2 days and thereafter at 2–4 weeks intervals to assess shunt position, regression of cyst size or resolution of hydrops. The follow-up scan was performed either in the two centers or in the referring outside centers. In these latter cases, the written reports were reviewed. Formal ethical review was not required as it was regarded as an audit of the outcome.

Statistical analysis was performed using Fisher’s exact test of the PSPP 2.0.1 statistical software. A *p* value < 0.05 was considered significant. Data are quoted as mean (range). Twelve of the cases were previously published in a multicenter series focusing on the surgical follow-up [15].

## Results

Twenty five fetuses with macrocystic CPAM were shunted with the Somatex^®^ shunt between 2016–2024 in two tertiary centers: 24 singletons and 1 affected fetus of a monochorionic-diamniotic twin pregnancy.

### Prenatal course

Mean gestational age at shunting was 24.7 (19–30) weeks. At the time of shunting 36.0% (9/25) fetuses presented with hydrops. In all cases, hydrops consisted exclusively of ascites accompanied by scalp oedema. Of the 9 cases with hydrops 5 were associated with polyhydramnios. Of the 16 fetuses without hydrops 4 had isolated ascites and the remaining 12 had a large lesion with a dominant cyst and severe mediastinal shift and 8 of these had polyhydramnios. CPAM was right sided in 64.0% (16/25) and left sided in 36.0% (9/25) (Table 1). All 9 fetuses with hydrops were associated with right-sided CPAM. Among the 4 cases with isolated ascites, 3 were right-sided and one left sided. No additional fetal anomalies were found during the first detailed ultrasound and the follow-up scans.

**Table 1:** Antenatal characteristics of fetuses with macrocystic CPAM

	*n* = 25
Right-sided CPAM	17 (64%)
Left-sided CPAM	8 (36%)
Hydrops fetalis	9 (36%)
Severe mediastinal shift	25 (100%)
Polyhydramnios	13 (52%)
Dominant cyst size	34 mm (18–50)
CVR	2.3 (1.1–4.3)

*CVR* CPAM volume to head circumference ratio

In the study period, TAS using the Somatex^®^ shunt was the first-line treatment in both centers, and no other shunt system was used. However, in two cases thoracocentesis of the macrocysts was performed prior to shunting. In one of these cases, thoracocentesis was performed in an outside center prior to referral for shunting and in the other case thoracocentesis was done because the fetus was in an unfavorable position for shunting and already hydropic. After thoracocentesis, the cysts refilled within a week and the Somatex^®^ shunt was successfully inserted.

Successful initial attempt of shunting was possible in 92% (23/25) cases. One shunt was falsely placed with the outer end of the shunt ending in the fetal subcutis and a successful second intervention was done in the same session. The other shunt dislocated immediately after shunt insertion intrathoracally, however, the patient rejected a reshunting and only the biggest cyst was punctured. On follow-up examinations, the lesion regressed partially.

There were two shunt dislocations in the further course of pregnancy: In one fetus, the outer end of the shunt dislocated intracutaneously, with new development of hydrothorax 2 days after the first shunting. Re-shunting was successfully performed. In another fetus the entire shunt dislocated into the amniotic cavity after 5 weeks. In this case, no reintervention was necessary as there was only minor reaccumulation of fluid in the lesion.

In one previous hydropic fetus, the ascites persisted despite successful TAS with regression of the lesion and regression of the scalp oedema. In this case peritoneo-amniotic shunting was performed. Hereafter the ascites resolved and there were no further complications during ongoing pregnancy. The repeated shunt insertions were not significantly associated with preterm birth or adverse outcome (*p* = 0.5).

All lesions showed regression of cysts size and mediastinal shift after shunting of the dominant cyst. Resolution of hydrops was observed in 89% (8/9) of the hydropic fetuses and occurred on average 4–5 weeks after shunting.

In one hydropic fetus shunted at 23 weeks, ipsilateral rib fractures became evident on ultrasound 4 weeks after shunt insertion. After birth, a fracture of 4 rips was confirmed. There were no maternal complications.

### Perinatal outcome

Overall survival rate to delivery was 100% (25/25). There were no terminations of pregnancy (TOP) and no intrauterine deaths (IUD). The mean gestational age at delivery was 38.3 (26–41) weeks. The mean interval from first shunting to delivery was 13.7 (5–19) weeks.

The preterm birth rate before 37 weeks was 20% (5/25). Preterm birth before 34 weeks occurred in one case, and the other 4 between the 35 and 37 weeks, including one planned delivery of monochorionic twins. The rate of premature rupture of membranes (PROM) was 12% (3/25), PROM occurred in a range of 5–11 weeks after shunt insertion (Table 2).

**Table 2:** Details of shunting

Mean GA at first shunting (weeks)	24.7 (19–30)
Successful first attempt of shunting	23 (92%
Reshunting	2 (8%)
Shunt dislocations	2 (8%)
PROM	3 (12%)
Preterm birth < 37 weeks	5 (20%)
Mean GA at delivery (weeks)	38.3 (26–41)
Survival	23 (92%)

*GA* gestational age, *PROM* premature rupture of membranes

A total of 64% (16/25) patients delivered vaginally, and caesarian section was done in the other cases for obstetrical reasons.

The overall survival rate was 92% (23/25). 94% (15/16) of the non-hydropic fetuses survived and 88.9% (8/9) of the hydropic fetuses. There were two neonatal deaths (NND): one occurred in a previously hydropic fetus born preterm at 26 weeks, 5 weeks after shunt insertion. Another child died at the age of 5 weeks because of sudden infant death after being successfully operated and discharged from the hospital. Hydrops did not significantly affect the survival rate (*p* = 0.13).

Data for the postpartum course were available for 23 patients; 2 patients were lost to follow-up after being born alive. Of the 23 cases with known long-term outcome, 22 underwent surgery during the initial postnatal hospital stay at a mean of 10.4 days (range 2–42 days). Only one child was operated after 10 months. All children underwent open surgery with lobectomy. Only one child underwent initially thoracoscopic removal of the intrathoracically dislocated shunt and open surgery with lobectomy 5 days thereafter.

**Fig. 2:**
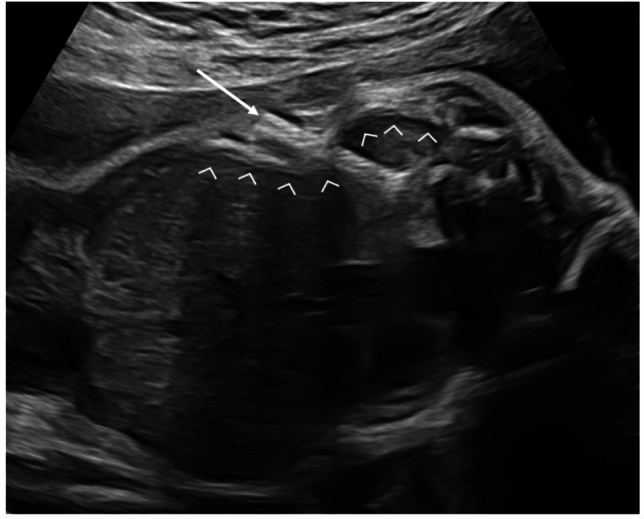
Somatex^®^ shunt (arrow) in a previously hydropic fetus at 26 weeks presenting with rip fractures (arrowheads) following shunt placement at 23 weeks

Skeletal anomalies were observed in 8/23 cases. One fetus had an intrauterine diagnosis of multiple rib fractures 4 weeks after shunt insertion (Fig. 2), which was confirmed after birth by *x* ray with additional scoliosis. Later in childhood, 7 children presented chest wall deformities, mostly pectus excavatum.

**Fig. 3:**
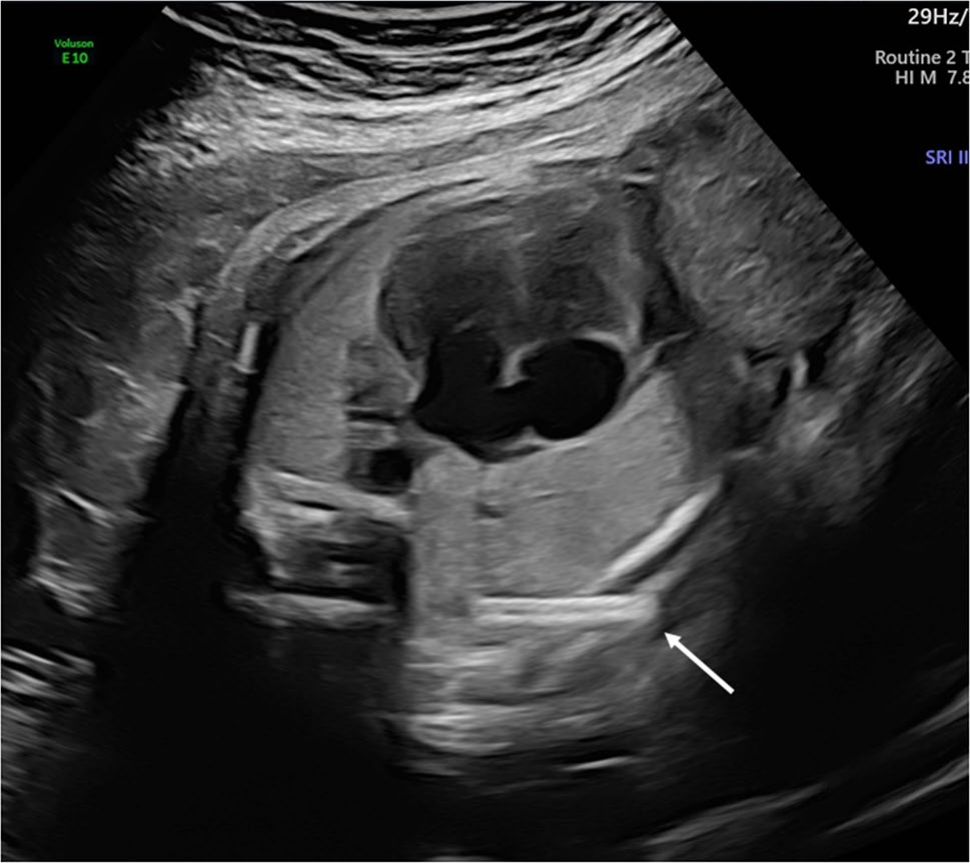
same fetus as in Fig. 1 at 39 weeks showing the dislodged shunt with the outer end embedded in the skin (arrow), note the reversal of the mediastinal shift and the complete drainage of the macrocysts despite the dislodged shunt

Of the 25 liveborns, 12 were delivered in the two study centers. Therefore, detailed information of the shunt position after birth was available in these cases: In one case, the (8.3%) shunt was dislodged in the amniotic cavity in the prenatal period, in 5 (42.7%) newborns the shunt was visible outside the skin and in the remaining 6 (50%) the shunt was covered with skin and therefore invisible. This condition could also be suspected on a prenatal ultrasound (Fig. 3).

## Discussion

Several shunt systems for TAS are available, and until now data only exist concerning the rocket shunt [8, 10, 12] and the Harrison fetal blader stent [11]. Since late 2014 the Somatex^®^ shunt is available and was primarily designed for vesicoamniotic shunting in fetal megacystis. As the Somatex^®^ shunt showed good performance in TAS for hydrothorax [14], we applied it for macrocystic CPAM since 2016. This is to our knowledge the first cohort of fetuses treated with the Somatex^®^ shunt for macrocystic CPAM. There is only one prior case report on shunting in CPAM using the Somatex^®^ shunt [16].

The mean gestational age at delivery with the Somatex^®^ shunt of 38 weeks is largely unchanged and rather reflects the high rate of induced labor to allow a planned delivery in a designated center than an effect of the shunt itself. For other types of shunts the reported mean gestational age at delivery ranges from 35–39 weeks [8, 9,10, 12]. Peranteau et al. [8] reported the lowest mean gestational age at birth of 35 weeks due to a preterm birth rate of 48%.

As in previous reports on shunting in CPAM, the regression rate of hydrops and of the lesion itself using the Somatex® shunt were high with 89% and 100%, respectively. Schrey et al. [12] found a 100% regression rate of size and hydrops, Peranteau et al. [8] reported on an 89% regression rate of hydrops. Both authors used the Rocket shunt. The survival rate with the Somatex^®^ shunt was equally high: 89% in hydropic cases and 94% in the absence of hydrops. In previous studies using different devices the survival rate in cases with hydrops ranged from 43–83 and 78–100% in fetuses without hydrops [8–12].

In 50% of the children born in our centers the Somatex^®^ shunt was embedded in the fetal subcutaneous layer and overgrown with skin at birth and was therefore invisible. Chan et al. [16] also reported a case of a Somatex^®^ shunt covered with skin at term birth in a fetus shunted at 27 weeks for macrocystic CPAM. This is also a known complication of TAS with the Somatex^®^ shunt for hydrothorax [14] and has never been described in the Harrison shunt. This is possibly due to the shorter length of the Somatex^®^ shunt (25 mm) in contrast to the longer usable length of 35 mm of the pigtail catheter and due to its straight design.

Chest wall deformities, especially Pectus excavatum which was observed later in childhood in 7/23 (30.4%) of our cohort, are known complications after surgery for lung malformations [10]. It seems that they are correlated to the lung malformation or lung hypoplasia itself and not to intrauterine TAS. Even children with congenital diaphragmatic hernia have a higher incidence of chest wall deformities [17]. In our cohort, there was only one case with already prenatally detected rib fractures that were confirmed in the neonatal period. Rib fractures as a complication of TAS occur especially if TAS is performed prior to 21 weeks [18]. Our case, however, had an uncomplicated shunt placement at 23 weeks. Although the causal relationship of the rip fractures and the shunt placement is unquestionable, the extent of damage does not correlate with the promptness of the intervention, that was well documented on video.

As skin lesions were reported in VAS for fetal megacystis in the first trimester with the Somatex^®^ shunt [19], we reviewed the shunted CPAM cases for this complication. But unlike in VAS, there were no skin lesions in TAS for macrocystic CPAM. The reason might be that shunting for fetal CPAM is done in higher weeks of pregnancy whereas shunting in fetal megacystis is frequently done before 14 weeks of pregnancy, when the fetal skin is immature. Unlike shunting for fetal megacystis, which is done anteriorly in the fetuses, shunting for CPAM is done mostly posteriorly so there is less risk for the fetus to get injured by the outer end of the shunt. Likewise, Grandt et al. [14] observed no skin lesion in shunting for fetal hydrothorax with the Somatex^®^ shunt.

As with other types of shunts, there were no maternal complications with the Somatex^®^ shunt [8, 10, 12]. Our work is not without limitations: As a retrospective two-center audit without a control group, our conclusions are necessarily limited to observational findings. The limited follow-up period precludes broad conclusions on the long-term respiratory and developmental outcomes. A future prospective or multicenter comparative study could help confirm our results. Although in our retrospective study, ethical approval was not requested by the board as it was an audit of outcome, ethical approval will be a prerequisite for further prospective and controlled studies.

## Conclusion

This study describes the first large cohort of fetuses undergoing TAS in macrocystic CPAM since the Somatex^®^ shunt became available in 2014. The technical success rate of TAS in macrocystic CPAM, the intrauterine course and the survival rates of hydropic and non-hydropic fetuses are comparable to other types of shunts. Therefore, the choice of the shunt in macrocystic CPAM can be made freely at the discretion of the physician in charge, the availability of shunt devices and economic factors. An advantage of the Somatex^®^ shunt is the smaller size of the delivering cannula which renders the placement more comfortable for the mother. However, clinicians should be aware that because of the short length of the Somatex^®^ shunt and its straight design it could be embedded in the subcutaneous tissue and not be seen outside the skin after birth, which complicates the removal.

## Data Availability

No datasets were generated or analysed during the current study.
